# Competitive Immunoplatform Based on Antibody-Functionalized Magnetic Nanoparticles for Label-Free Cortisol Detection Using Si_3_N_4_ Ion-Sensitive Field-Effect Transistors

**DOI:** 10.3390/bios16100561

**Published:** 2026-10-04

**Authors:** Hamdi Ben Halima, Nadia Zine, Joan Bausells, Abdelhamid Errachid, Nicole Jaffrezic-Renault

**Affiliations:** 1 Institut UTINAM (UMR CNRS 6213), Université Marie et Louis Pasteur, F-25000 Besançon, France; 2 Institut de Sciences Analytiques (ISA)-UMR 5280, Université Claude Bernard Lyon 1, 5 Rue de la Doua, 69100 Lyon, France; nadia.zine@univ-lyon1.fr (N.Z.); abdelhamid.errachid-el-salhi@univ-lyon1.fr (A.E.); 3 Institute of Microelectronics of Barcelona (IMB-CNM, CSIC), Campus UAB, 08193 Bellaterra, Spain; joan.bausells@imb-cnm.csic.es

**Keywords:** cortisol, FET, magnetic nanoparticles, competitive immunoassay, electrochemical biosensor, label-free detection, point-of-care diagnostics, stress monitoring, salivary biomarkers

## Abstract

Cortisol is a key biomarker involved in the regulation of the hypothalamic–pituitary–adrenal axis and is widely used for assessing physiological stress and endocrine disorders. However, its small molecular size and low charge make its direct detection using field-effect transistor (FET)-based biosensors challenging. In this work, we present a competitive ImmunoFET biosensing platform combining a Si_3_N_4_-gated ion-sensitive field-effect transistor (ISFET) with anti-cortisol polyclonal antibody-functionalized magnetic nanoparticles (MNPs) for label-free cortisol detection. The sensing strategy relies on a competitive immunoassay in which cortisol in the sample competes with cortisol immobilized on the ISFET surface for antibody binding sites on the MNPs. Following a 45 min pre-incubation, magnetic separation, and redispersion, the MNPs were brought into contact with the cortisol-functionalized FET and analyzed by electrochemical impedance spectroscopy (EIS). The charge-transfer resistance decreased from 4.012 MΩ to 2.599 MΩ as the cortisol concentration increased from 0.02 to 0.6 ng/mL, confirming the expected competitive behavior. The sensor exhibited a concentration-dependent response over the 0.02–0.6 ng/mL cortisol range, with a limit of detection of 0.006 ng/mL. The platform also showed a stronger response toward cortisol than the investigated non-target biomarkers, including NT-proBNP, TNF-α, and IL-10. Its applicability was further demonstrated in real saliva using the standard addition method, yielding a cortisol concentration of 11.6 ng/mL, compared with an expected value of 10.2 ng/mL, corresponding to a 113.7% recovery. These preliminary results demonstrate the feasibility of the proposed MNP-assisted competitive FET for cortisol detection in biological samples and provide a basis for further development toward point-of-care applications.

## 1. Introduction

Cortisol is the primary glucocorticoid hormone secreted by the adrenal cortex and plays a central role in the regulation of metabolism, immune and inflammatory responses, cardiovascular function, and adaptation to stress through the hypothalamic–pituitary–adrenal (HPA) axis [1,2]. Dysregulation of cortisol secretion or action has been associated with a range of pathological conditions, including Cushing’s syndrome, Addison’s disease, depression, anxiety disorders, metabolic syndrome, and cardiovascular diseases [3,4].

Because cortisol follows a pronounced circadian rhythm and responds dynamically to both physiological and psychological stress, it is widely recognized as an important biomarker for stress monitoring and personalized healthcare applications [5,6,7]. In recent years, non-invasive biofluids, such as saliva, sweat, and interstitial fluid, have attracted increasing attention for cortisol monitoring, offering opportunities for minimally invasive and potentially continuous health assessment [4,5]. Among these biofluids, salivary cortisol is particularly relevant because it reflects the biologically active, unbound fraction of circulating cortisol. In parallel, sweat-based sensing has enabled the development of wearable aptasensors for real-time cortisol monitoring [8,9].

Conventional cortisol measurement techniques, including enzyme-linked immunosorbent assay (ELISA), radioimmunoassay (RIA), high-performance liquid chromatography (HPLC), and liquid chromatography–tandem mass spectrometry (LC–MS/MS), provide high sensitivity and specificity. However, these methods generally require centralized laboratory facilities, specialized equipment, trained personnel, and relatively time-consuming sample processing, limiting their applicability to real-time and point-of-care monitoring [10,11,12,13]. These limitations have stimulated the development of portable, decentralized, and label-free biosensing technologies for cortisol detection.

Among emerging sensing platforms, field-effect transistor (FET)-based biosensors have attracted considerable interest because of their label-free operation, high sensitivity, rapid response, low power consumption, miniaturization, and compatibility with semiconductor fabrication technologies [8,9]. Since their introduction by Bergveld, ISFETs have been extensively investigated for detecting chemical and biological species by transducing changes at the gate/electrolyte interface into measurable electrical signals [14,15,16,17].

Despite these advantages, the direct detection of cortisol using BioFET platforms remains challenging because cortisol is a small, essentially neutral steroid molecule, and its molecular recognition does not necessarily produce a large intrinsic change in charge. Consequently, the electrical transduction of cortisol binding can be weak and strongly dependent on the receptor architecture and interfacial environment [18,19]. Moreover, Debye screening in physiological media can limit the effective sensing distance between the recognition event and the FET surface, thereby reducing the sensitivity of label-free detection configurations [17,19].

To address these limitations, competitive immunoassay formats are particularly well suited to the detection of small molecules such as cortisol, for which conventional sandwich immunoassays are generally difficult to implement because the analyte provides limited opportunities for simultaneous binding of two antibodies [11,20]. In a competitive immunoassay, free cortisol competes with an immobilized cortisol derivative or labeled cortisol conjugate for a limited number of antibody binding sites, resulting in an analytical signal that is related to the cortisol concentration in the sample [11,20]. The combination of competitive immunoassays with electrochemical transducers has demonstrated promising analytical performance for cortisol detection [21,22]. FET-based approaches have also been investigated for cortisol sensing, although their implementation requires careful consideration of the receptor architecture and interfacial signal-transduction mechanism [19].

Furthermore, magnetic nanoparticles (MNPs) have emerged as valuable components in biosensing strategies because of their high surface-area-to-volume ratio, versatile surface functionalization, and magnetic manipulability [23,24]. Antibody-functionalized MNPs can facilitate efficient target recognition and capture, followed by magnetic separation and analyte preconcentration. These properties can enhance sensitivity and selectivity while helping to reduce matrix-related interferences in complex biological samples [25].

Wearable and point-of-care cortisol biosensors have received increasing attention for real-time stress monitoring and personalized healthcare applications. Nevertheless, several challenges, including biofouling, matrix-dependent effects, signal drift, reproducibility, and long-term operational stability, continue to limit their practical and clinical translation [11,26]. Therefore, the development of robust sensing architectures that integrate selective biorecognition, efficient signal transduction, and appropriate sample-preparation strategies remains necessary.

**Figure 1:**
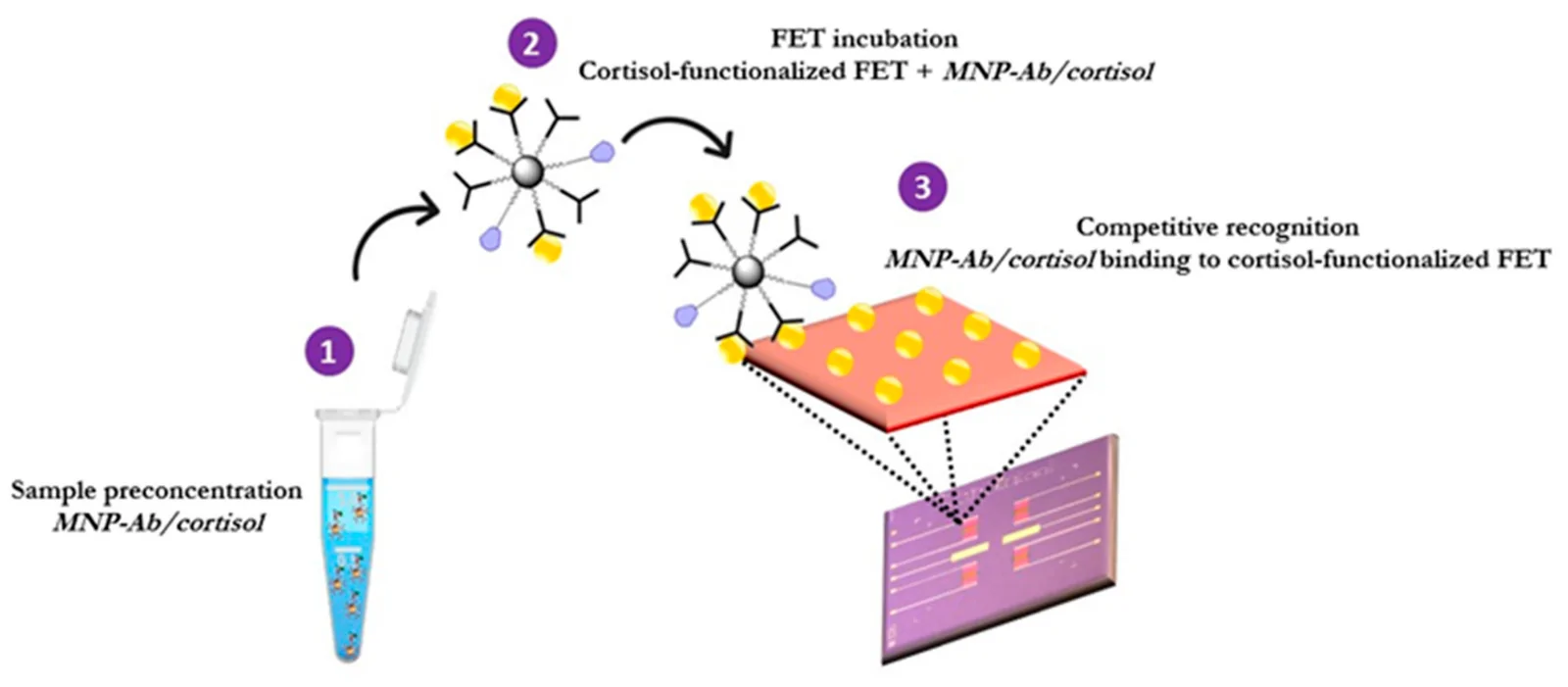
Scheme of the competitive approach for cortisol detection. (**1**) After the sample preconcentration step using MNP-Ab/cortisol; (**2**) the cortisol-functionalized FET is incubated in the PBS solution containing the MNP-Ab/cortisol; (**3**) recognition of the cortisol-functionalized FET by MNP-Ab/cortisol.

In this work, we propose a competitive immunoplatform based on a Si_3_N_4_-gated ion-sensitive field-effect transistor (ISFET) combined with antibody-functionalized magnetic nanoparticles for cortisol detection. As schematically illustrated in Figure 1, the proposed strategy integrates a magnetic preconcentration step with a competitive immunoassay performed at the cortisol-functionalized ISFET surface. Briefly, cortisol present in the sample is first captured by the antibodies immobilized on the magnetic nanoparticles, allowing its preconcentration and isolation from the sample matrix. The resulting MNP-Ab/cortisol complexes are then incubated with the ISFET working electrode functionalized with immobilized cortisol. A competitive interaction occurs between the cortisol molecules captured by the magnetic nanoparticles and the surface-immobilized cortisol for the available antibody binding sites. Consequently, the amount of antibody remaining available to interact with the cortisol-functionalized ISFET surface depends on the cortisol concentration in the sample, resulting in a concentration-dependent electrical response. This competitive configuration enables label-free cortisol detection while combining magnetic preconcentration with electrical transduction at the ISFET surface.

## 2. Materials and Methods

### 2.1. Materials and Chemicals

Hydrocortisone (cortisol, 99% purity, Cat. No. ab141250) was purchased from Abcam (Rosny-sous-Bois, France). TNF-α (Cat. No. 210-TA) and IL-10 (Cat. No. 1064-IL) were obtained from R&D Systems (Noyal-Châtillon-sur-Seiche, France), whereas NT-proBNP (Cat. No. 8NT2) was supplied by HyTest (Turku, Finland). Polyclonal anti-human TNF-α antibody (Cat. No. AF-410-SP) and polyclonal anti-human IL-10 antibody (Cat. No. AF-217-SP) were obtained from R&D Systems (France). Polyclonal anti-cortisol antibody produced in sheep (Cat. No. PA1-85347) was purchased from Invitrogen (Thermo Fisher Scientific, Lyon, France). Polyclonal anti-human NT-proBNP antibody (Cat. No. RD184486100) was purchased from BioVendor (Brno-Řečkovice, Czech Republic). Phosphate-buffered saline (PBS) tablets (MFCD00131855), ethanolamine (purity ≥ 98%), and absolute ethanol (purity 95.0%, CAS No. 64-17-5) were purchased from Sigma-Aldrich (Saint-Quentin-Fallavier, France). 11-Triethoxysilylundecanal (TESUD, 90%, CAS No. 116047-42-8) was obtained from ABCR (Karlsruhe, Germany). Bis (sulfosuccinimidyl) suberate (BS3, CAS No. 82436-77-9) was purchased from Thermo Fisher Scientific (Waltham, MA, USA). Ultrapure water (resistivity > 18 MΩ·cm) was produced using an Elga PURELAB Classic system (Veolia, France). The PBS buffer was prepared by dissolving PBS tablets in ultrapure water according to the manufacturer’s instructions, yielding a 0.01 M phosphate buffer (pH 7.4) containing 0.0027 M potassium chloride and 0.137 M sodium chloride. EPO-TEK H70E2LC epoxy resin (Parts A and B) was supplied by Epoxy Technology Inc. (Orgeval, France).

### 2.2. ISFET Device Fabrication

The ISFET devices were fabricated at the National Microelectronics Centre (CSIC, Institute of Microelectronics of Barcelona) following a previously reported microfabrication process [27,28,29,30,31,32]. Briefly, 100 mm diameter silicon wafers with a boron substrate doping of 1015 cm^−3^ were used to fabricate the N-type FETs. The source and drain regions were formed by phosphorus ion implantation (dose of 4.2⋅1015 cm^−2^), followed by standard photolithography and etching processes. The gate dielectric consisted of a 78 nm thick silicon dioxide layer and a 100 nm thick silicon nitride layer deposited by LPCVD. The silicon nitride layer was patterned to define the gate region, while the electrical contacts were formed using titanium/platinum metal interconnects with thicknesses of 15 nm and 150 nm, respectively. A passivation layer consisting of 400 nm SiO_2_ and 400 nm Si_3_N_4_ was subsequently deposited. Four ISFETs and two Ag/AgCl reference microelectrodes were integrated at the center of an 8060 × 8440 mm^2^ silicon chip. The gate dimensions were 20 μm in width and 400 μm in length, with a gate dielectric thickness of Si_3_N_4_ (100 nm)/SiO_2_ (80 nm).

The fabricated chip was mounted onto a printed circuit board (PCB) using epoxy resin. The ISFET contact pads were electrically connected to the PCB using 25 μm-diameter aluminum wire by wire bonding. The wire bonding procedure was performed using 25 μm-diameter aluminum wire with a Kulicke & Soffa 4523A digital wire bonder (Singapore). The bonding area, wires, and gold tracks of the PCB were subsequently protected with epoxy resin and cured at 150 °C for 5 min. Further details of the ISFET fabrication process have been reported previously [27,31].

### 2.3. Synthesis and Characterization of MNPs

The magnetic latex nanoparticles (MNPs) were prepared following the synthesis procedure previously reported in [32]. Briefly, magnetic latex seed particles were first prepared by polymerization in the presence of divinylbenzene and potassium persulfate, followed by functionalization with an amino-containing dextran polymer. The amino-dextran was adsorbed onto the particle surface to introduce amine groups suitable for subsequent antibody immobilization. The resulting functionalized MNPs were characterized by transmission electron microscopy (TEM) and dynamic light scattering (DLS), confirming their core–shell structure and submicron size. The average hydrodynamic diameter was approximately 302 nm, with a narrow size distribution. Detailed synthesis and characterization procedures have been reported previously [32].

### 2.4. Sample Preparation

Saliva was collected from a healthy volunteer using a synthetic swab (Salivette^®^, Sarstedt, Germany). The human samples were collected in the morning (10 o’clock) with informed consent from the healthy volunteers based on the Declaration of Helsinki. Briefly, the volunteer was instructed to move the swab throughout the oral cavity for 2 min at a self-selected pace. The saliva sample was subsequently recovered by centrifugation of the swab at 7000 rpm for 5 min at 4 °C, as previously reported [31]. The recovered saliva was spiked with cortisol at a concentration of 7.5 ng/mL. Cortisol quantification was performed using the standard addition method. Briefly, 50 μL of saliva was transferred into each of four 1.5 mL Eppendorf^®^ Lo-Bind centrifuge tubes (Eppendorf, Montesson, France). The first tube was diluted to a final volume of 1 mL by adding 950 μL of PBS. The remaining three tubes were supplemented with 10, 20, and 50 μL of a 20 ng/mL cortisol standard solution, respectively, and then brought to a final volume of 1 mL with PBS. All standard addition solutions were analyzed in quadruplicate (*n* = 4). EIS measurements were performed according to the procedure described in the following section. Additionally, the cortisol concentration was controlled through HPLC-ESI-Q-ToF analyses performed with a 1200 Infinity HPLC (Agilent Technologies, Les Ulis, France) coupled to a 6530 Infinity Jet Stream ESI-Q-ToF (Agilent Technologies) as in [33].

### 2.5. Biofunctionalization of the FET

The biofunctionalization procedure was applied to each biomarker and consisted of several steps. First, the ISFET chips were cleaned by sonication in acetone and thoroughly rinsed with Milli-Q water. The devices were subsequently treated with a UV/Ozone Procleaner™ (BioForce, Konstanz, Germany) to activate the surface and generate reactive groups for subsequent silane functionalization. TESUD (11-triethoxysilyl undecanal) was then deposited onto the activated surface using a vapor-phase process. The chips were subsequently placed in an oven at 100 °C for 1 h, followed by rinsing with pure ethanol and drying under a nitrogen stream. The functionalized devices were then incubated with a 10 µg/mL solution of the corresponding target antigen. Finally, to minimize nonspecific interactions during the detection step, the remaining reactive sites were blocked by incubation with 1% ethanolamine in PBS for 20 min at room temperature (20 ± 2 °C). The surface morphology of the MNPs immobilized on the ISFET was examined by scanning electron microscopy (SEM, Thermo Fisher Scientific, Lyon, France) using an FEI Quanta FEG 250 microscope.

### 2.6. MNPs Biofunctionalization and Antigen Preconcentration

First, 10 µL of the mother solution containing MNPs was washed three times with 200 µL of 10 mM PBS (pH 7.4). A magnetic rack was used to immobilize the magnetic nanoparticles during removal of the supernatant and addition of fresh PBS. The amine groups present on the MNP surface were subsequently activated by incubation with 200 µL of BS_3_ (10 µg/mL) at room temperature (20 ± 2 °C) under gentle stirring for 10 min. The excess BS_3_ was then removed, and the MNPs were washed twice with 200 µL of PBS. Subsequently, 100 µL of purified biomarker-specific polyclonal antibody was added to the activated MNPs suspension. The MNPs–antibody (MNPs-Ab) mixture was gently stirred at room temperature (20 ± 2 °C) for 3 h.

Following antibody immobilization, the MNPs-Ab complexes were magnetically separated using a magnetic rack, and the supernatant was removed. The remaining reactive sites were blocked by adding 500 µL of 1% ethanolamine in PBS to each Eppendorf tube. The suspension was stirred at 33 rpm and room temperature for 30 min. The excess ethanolamine was subsequently removed, and the complexes were washed twice with 1 mL of 10 mM PBS (pH 7.4) using the magnetic rack for nanoparticle separation.

For antibody functionalization optimization, polyclonal antibody concentrations of 1, 5, and 10 µg/mL were investigated. For each concentration, the corresponding antibody-functionalized MNPs suspension was incubated with the ImmunoFET, followed by electrochemical impedance spectroscopy (EIS) measurements. For antigen preconcentration experiments, the MNPs-Ab complexes were incubated with the target antigen at concentrations of 0.02, 0.04, 0.1, 0.2, 0.4, and 0.6 ng/mL for 45 min. Then, EIS measurements were carried out to characterize the ImmunoFETs after each concentration.

### 2.7. Electrochemical Measurements

All experiments were conducted at room temperature (20 ± 2 °C) inside a Faraday cage. A platinum counter electrode and an Ag/AgCl reference electrode were supplied by BVT Technologies (Strážek, Czech Republic). Electrochemical impedance spectroscopy (EIS) measurements were carried out using a VMP3 multichannel potentiostat (BioLogic-EC-Lab, Seyssinet-Pariset, France), while data acquisition and modeling were performed using EC-Lab software (version 11.30, BioLogic, Seyssinet-Pariset, France). Electrochemical impedance spectroscopy (EIS) measurements were performed in 7 mL of 1× PBS over a frequency range from 10 kHz to 10 Hz, using two frequency points per decade. A sinusoidal AC perturbation of 75 mV was applied without imposing a DC bias potential, with E_dc_ set to 0 V versus the Ag/AgCl reference electrode.

The experimental impedance spectra were analyzed using this equivalent electrical circuit [R_1_ + C_dl_/R_2_]. In this model, R_1_ represents the uncompensated resistance of the PBS electrolyte, whereas C_dl_ corresponds to the double-layer capacitance associated with the interfacial behavior. R_2_, corresponding to the charge-transfer resistance (R_ct_), was used as the main parameter for evaluating the sensor response.

## 3. Results and Discussion

### 3.1. Coupling of the Immunomagnetic Preconcentration Strategy with the ET

The integration of antibody-functionalized MNPs with the FET was investigated to enhance the interaction between the biorecognition elements and the sensing interface. The present strategy relies on a competitive immunoassay format for cortisol detection The sensing principle is based on the competition between free cortisol in the sample and cortisol immobilized on the ImmunoFET gate for the binding sites of the anti-cortisol polyclonal antibodies grafted onto the MNPs.

To assess the ability of the functionalized MNPs to interact with the cortisol-modified sensing surface, the FET was incubated with anti-cortisol antibody-functionalized MNPs under the optimized conditions. After incubation, the sensor was thoroughly rinsed with PBS to remove loosely associated nanoparticles. The resulting sensing interface was subsequently examined by scanning electron microscopy (SEM).

**Figure 2:**
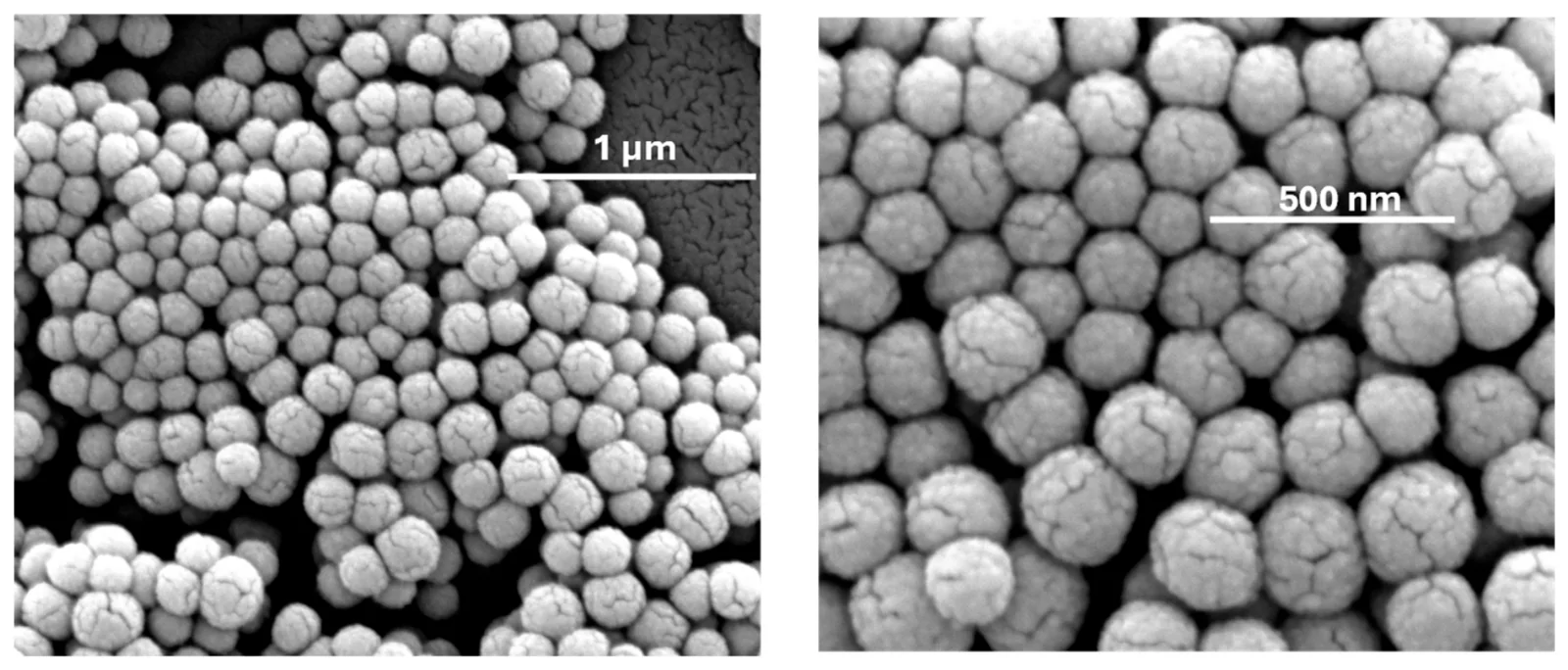
SEM images of the cortisol-functionalized FET after incubation with anti-cortisol antibody-functionalized MNPs, showing MNP retention at the sensing interface.

The SEM images provide morphological evidence of MNP retention at the FET gate following the incubation and washing steps. The presence of nanoparticles on the cortisol-functionalized sensing area indicates that the anti-cortisol antibodies immobilized on the MNPs recognize the cortisol molecules present at the gate surface, as shown in Figure 2. This observation confirms the effective coupling between the immunomagnetic component and the ET interface.

The observed MNP retention is particularly relevant to the competitive detection mechanism. In the absence of, or at low concentrations of, free cortisol, the antibody binding sites on the MNPs remain available to interact with the cortisol immobilized on the gate, resulting in a higher amount of MNPs associated with the sensing surface. As the concentration of free cortisol increases, the antibody binding sites become progressively occupied by cortisol in solution, limiting the subsequent interaction of the MNPs with the cortisol-functionalized gate. This competition is therefore expected to produce a concentration-dependent change in the interfacial impedance.

Overall, these observations support the feasibility of combining the immunomagnetic preconcentration step with the cortisol-functionalized FET and provide morphological evidence for the antibody–cortisol recognition occurring at the sensor interface. The analytical performance of this competitive configuration was subsequently evaluated by monitoring the impedance response over an increasing range of cortisol concentrations.

### 3.2. Optimization of Antibody Functionalization of MNPs

**Figure 3:**
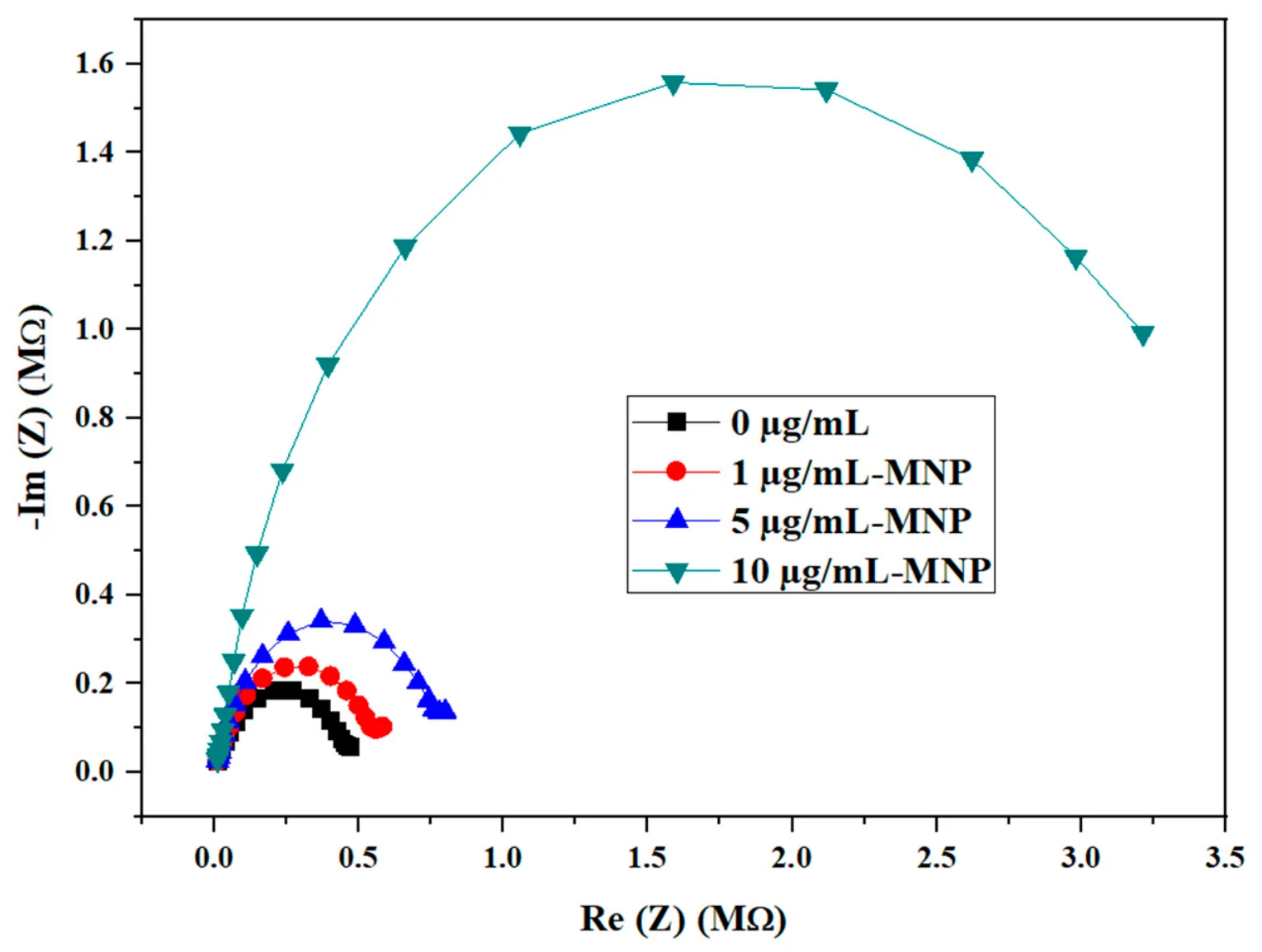
Nyquist plots of the cortisol-functionalized FET incubated with MNPs functionalized with different concentrations of anti-cortisol polyclonal antibody.

The concentration of polyclonal antibody used for MNPs functionalization was optimized to maximize the interaction between the MNP–antibody complexes and the cortisol-functionalized FET. MNPs functionalized with polyclonal antibody at concentrations of 1, 5, and 10 µg/mL were evaluated. After biofunctionalization, the resulting MNP–Ab complexes were incubated with the antigen-functionalized FET, and the corresponding electrochemical impedance responses were recorded by EIS. The Nyquist plots obtained under the different experimental conditions are shown in Figure 3.

The unmodified condition exhibited a relatively small semicircular response, whereas the introduction of antibody-functionalized MNPs resulted in a progressive increase in the impedance response. At 1 µg/mL antibody, the MNP–Ab complexes produced a moderate increase in the diameter of the semicircle. A further increase was observed when the antibody concentration was raised to 5 µg/mL, indicating a stronger interaction between the MNP–Ab complexes and the antigen present at the FET interface.

A markedly higher impedance response was obtained when the MNPs were functionalized using 10 µg/mL antibody. In this case, the Nyquist plot exhibited a substantially larger semicircle than those obtained at 1 and 5 µg/mL. The real impedance reached approximately 3.2 MΩ, compared with approximately 0.6 and 0.8 MΩ for the 1 and 5 µg/mL conditions, respectively. This pronounced increase indicates a stronger modification of the interfacial properties of the cortisol-functionalized FET following incubation with the MNP–Ab complexes.

The enhanced impedance response observed with increasing antibody concentration can be attributed to the higher density of antibody molecules available on the MNP surface. This increases the probability of antibody–antigen recognition at the sensor interface and consequently promotes a greater accumulation of MNP–Ab complexes on the cortisol-functionalized FET. The resulting modification of the interfacial layer produces a significant change in the impedance response.

Among the concentrations investigated, 10 µg/mL provided the highest impedimetric response and was therefore selected for subsequent antigen preconcentration experiments. This concentration was subsequently used for the incubation of MNP–Ab complexes with increasing concentrations of the target antigen.

### 3.3. Cortisol Detection in Standard Solutions

The analytical response of the competitive assay was evaluated using cortisol standard solutions over the concentration range of 0.02–0.6 ng/mL. Anti-cortisol antibody-functionalized MNPs, prepared under the optimized conditions (10 µg/mL antibody), were first incubated with the cortisol standards for 45 min. After magnetic separation and redispersion in PBS, the resulting MNP suspensions were brought into contact with the cortisol-functionalized FET and characterized by EIS.

**Figure 4:**
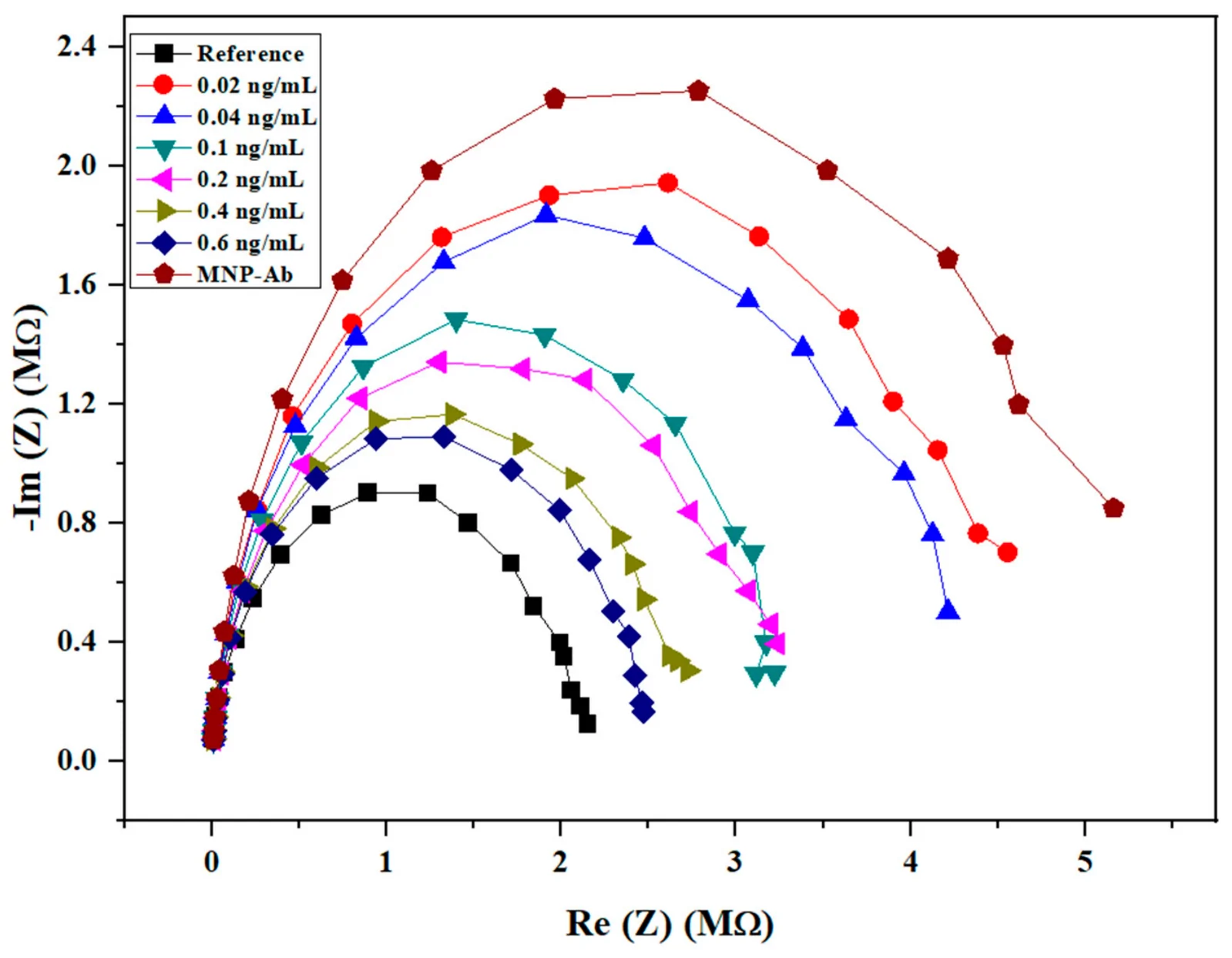
Nyquist plots obtained for the competitive detection of cortisol using the FET after incubation with cortisol-loaded, antibody-functionalized MNPs.

The Nyquist plots obtained at the different cortisol concentrations are shown in Figure 4. A clear concentration-dependent evolution of the impedance response was observed. The semicircle diameter progressively decreased as the cortisol concentration increased, indicating a reduction in the interfacial charge-transfer resistance. This behavior is consistent with the proposed competitive mechanism.

The fitted parameters are summarized in Table 1. The Rct value decreased from 4.012 MΩ at 0.02 ng/mL to 3.722, 3.298, 2.967, 2.695, and 2.599 MΩ at 0.04, 0.1, 0.2, 0.4, and 0.6 ng/mL, respectively. The progressive decrease in Rct with increasing cortisol concentration demonstrates that the concentration of the analyte directly influences the amount of MNP–Ab complexes retained at the sensing interface.

This trend can be explained by the competitive recognition mechanism. At low cortisol concentrations, a large fraction of the antibody binding sites on the MNPs remain available after the preconcentration step. These unoccupied sites can subsequently interact with the cortisol immobilized on the FET gate, resulting in greater MNP retention and a larger interfacial impedance. When the cortisol concentration increases, more antibody binding sites are occupied by free cortisol in solution. Consequently, fewer sites remain available to interact with the immobilized cortisol on the gate, leading to lower MNP retention and a reduced Rct value.

The MNP–Ab condition exhibited the highest Rct value (4.935 MΩ), whereas the reference FET showed an Rct value of 2.135 MΩ. The higher resistance observed after incubation with MNP–Ab is consistent with the strong modification of the sensing interface produced by nanoparticle retention. Importantly, the concentration-dependent decrease in Rct observed after exposure to cortisol is in agreement with the inverse response expected for a competitive immunoassay.

Although the response remains clearly concentration-dependent across the investigated range, the difference between 0.4 and 0.6 ng/mL is relatively small, suggesting that the response may begin to approach a plateau at the upper end of the tested range. Additional concentrations and replicate measurements would therefore be required to establish the linear or nonlinear calibration model, analytical sensitivity, limit of detection, and reproducibility of the sensor.

### 3.4. Selectivity Study

**Figure 5:**
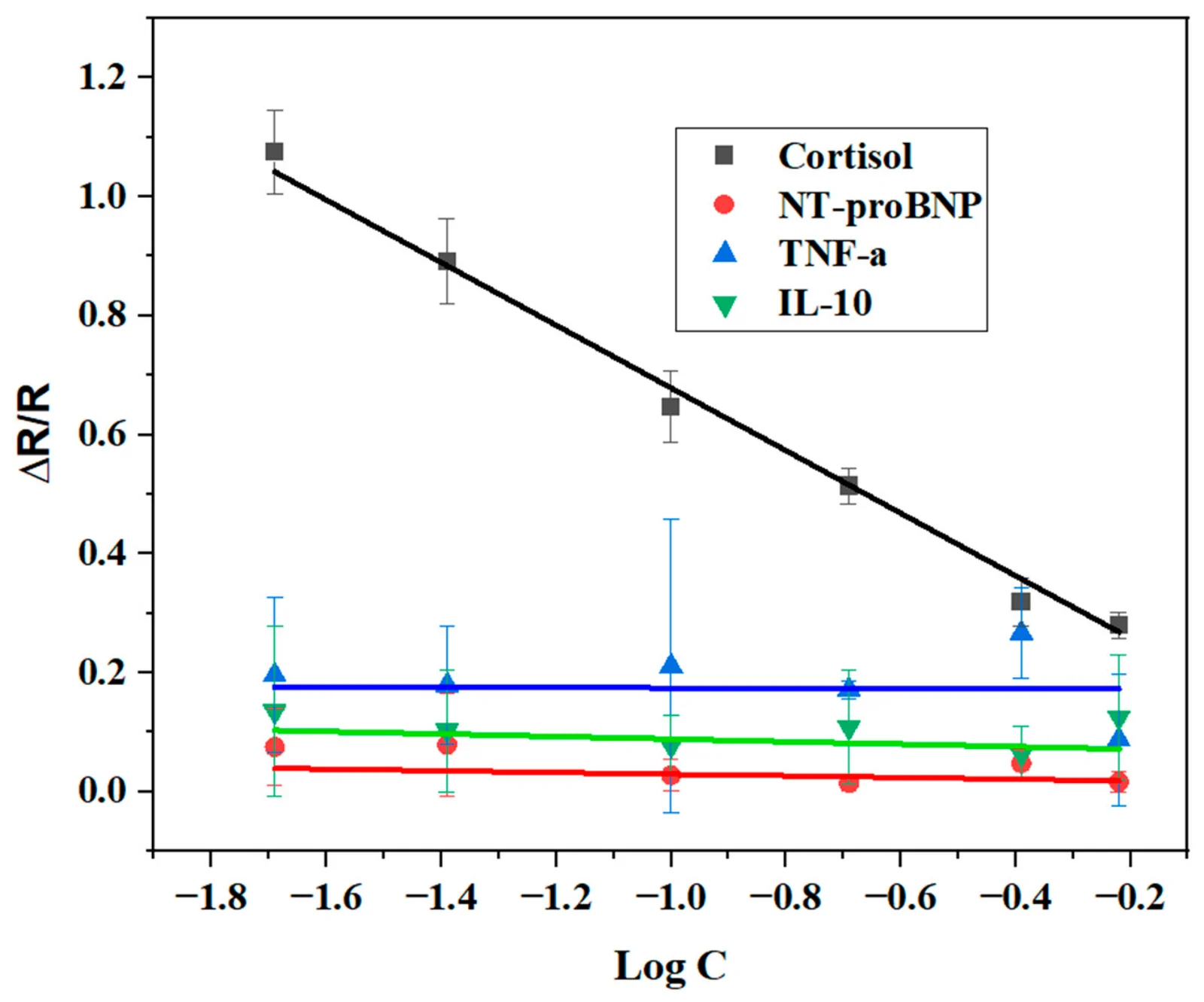
Relative impedance response of the competitive FET for cortisol and selected non-target biomarkers (NT-proBNP, TNF-α, and IL-10).

The selectivity of the proposed platform was evaluated by comparing the impedance responses obtained for cortisol with those obtained for the non-target salivary biomarkers NT-proBNP, TNF-α, and IL-10. As shown in Figure 5, cortisol exhibited a substantially stronger and more systematic response than the investigated non-target biomarkers over the tested concentration range. The calibration curve obtained for cortisol showed a clear linear dependence, described by the equation Y = −0.526x + 0.152, with an excellent coefficient of determination (R^2^ = 0.99). In contrast, the responses obtained for NT-proBNP, TNF-α, and IL-10 showed very weak concentration-dependent variations, with calibration slopes of −0.014, −0.002, and −0.021, respectively, and low coefficients of determination (R^2^ = 0.29, 0.09, and 0.15, respectively). These results indicate that the impedance response of the proposed platform is strongly dependent on cortisol concentration, whereas the non-target biomarkers produce only minor and poorly correlated responses. The markedly stronger and more reproducible response observed for cortisol can be attributed to the specific interaction between cortisol and the anti-cortisol polyclonal antibodies immobilized on the magnetic nanoparticles (MNPs). In the competitive configuration, cortisol present in the sample is captured by the antibody-functionalized MNPs, and the resulting MNP–Ab/cortisol complexes interact with the cortisol-functionalized FET surface. In contrast, NT-proBNP, TNF-α, and IL-10 do not undergo the same specific recognition process and therefore result in substantially lower retention of MNP–Ab complexes at the cortisol-functionalized FET interface. The low slopes and R^2^ values obtained for these non-target biomarkers further support the absence of a significant concentration-dependent response under the experimental conditions investigated.

The analytical performance of the proposed FET was compared with that of previously reported electrochemical immunosensors for cortisol detection (Table 2). The developed Si_3_N_4_-based FET achieved a detection limit of 0.006 ng/mL over the concentration range of 0.02–0.6 ng/mL. The limit of detection (LOD) of the proposed sensor was estimated using LOD = 3 S/m [34], where S is the residual standard deviation of the linear regression, and m is the slope of the regression line. These results demonstrate the capability of the proposed platform to detect cortisol at sub-ng/mL concentrations and show performance comparable to that of previously reported cortisol sensors. The integration of antibody-functionalized magnetic nanoparticles provides an additional immunomagnetic preconcentration step, while the Si_3_N_4_-based FET enables compact, label-free impedimetric detection. The proposed system shows promising potential for low-level cortisol monitoring and point-of-care applications.

### 3.5. Determination of Cortisol in Real Saliva Using the Standard Addition Method

The applicability of the proposed competitive FET was further evaluated using a saliva sample. Cortisol quantification was performed using the standard addition method, and the samples were analyzed in quadruplicate (*n* = 4) by EIS under the optimized experimental conditions. The relative impedance response (*ΔR*/*R*) was plotted as a function of the added cortisol concentration, and the experimental data were fitted using a four-parameter logistic (4PL) model.

**Figure 6:**
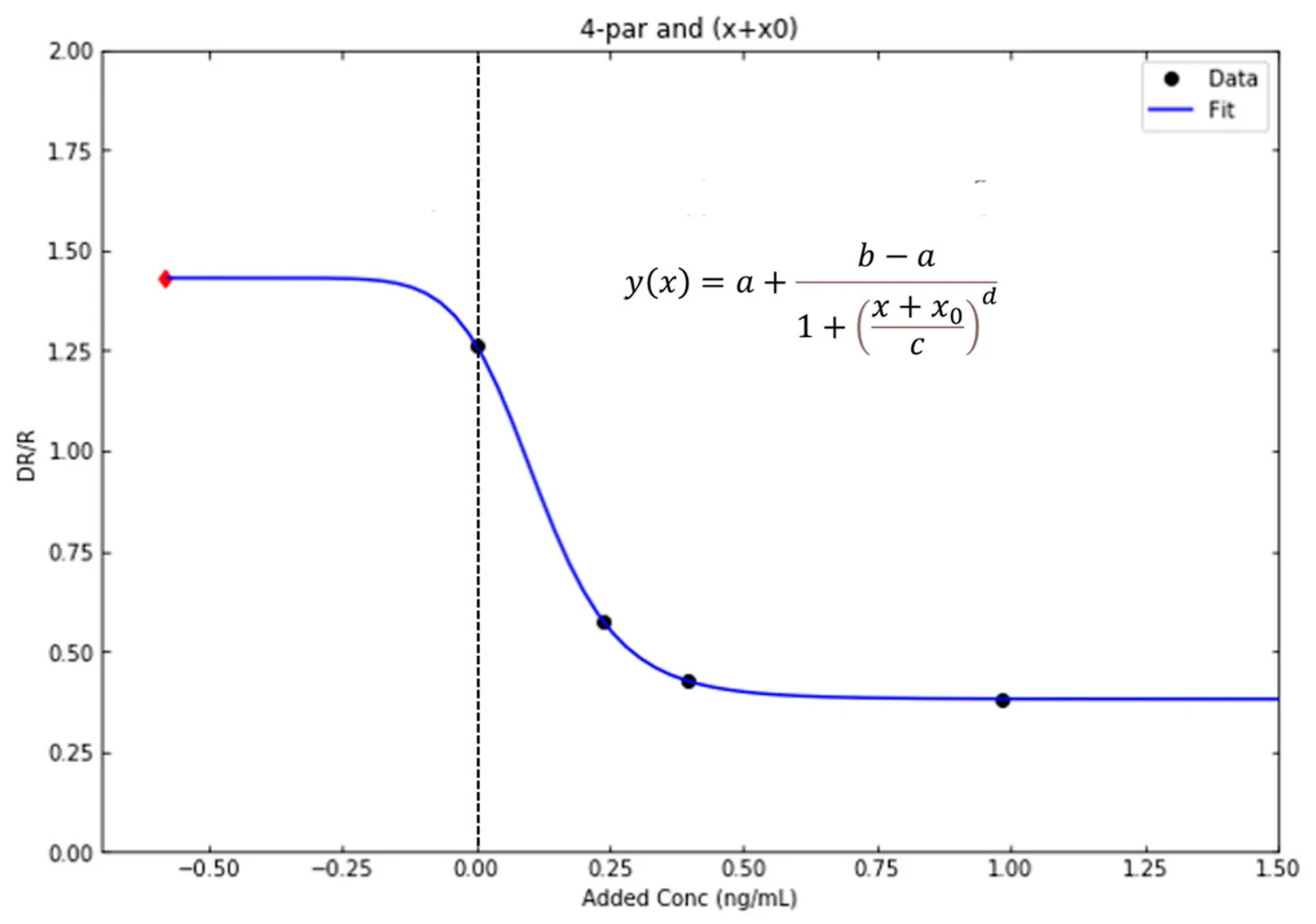
Four-parameter logistic fit of the relative impedance response as a function of the added cortisol concentration in saliva.

As shown in Figure 6, the relative impedance response exhibited a concentration-dependent variation with increasing cortisol additions, in agreement with the competitive sensing mechanism. By using the 4PL equation shown in the inset of Figure 6, the curve is shifted to the left by *x*_0_, which is the unknown value of the concentration in the saliva sample. The sample at *x* = *−x*_0_ corresponds to a zero-cortisol concentration, for which we know the ΔR/R value, and this gives the “*b*” parameter directly, because from the equation we get *y(−x*_0_) = *b*. The remaining four parameters, *a*, *c*, *d*, *x*_0_, can be calculated by fitting the experimental points to the 4PL model, which can be done numerically. The result is shown in Figure 6. The value of the *x*_0_ parameter is 0.58 ng/mL, and it represents the cortisol concentration in the diluted saliva sample used for the standard addition measurements. Considering the dilution factor of 20, the cortisol concentration in the original saliva sample was calculated as follows:

Cortisol concentration = 0.58 × 20 = 11.6 ± 0.5 ng/mL

The saliva sample used in this experiment had previously been characterized and contained 2.7 ng/mL of endogenous cortisol [31]. In the present experiment, 7.5 ng/mL of cortisol was additionally introduced into this saliva sample. Therefore, the expected total cortisol concentration was 10.2 ng/mL (2.7 ng/mL endogenous cortisol + 7.5 ng/mL added cortisol). The concentration determined by the proposed FET (11.6 ng/mL) was close to the expected value, with a difference of 1.4 ng/mL. This corresponds to a recovery of approximately 113.7%, demonstrating the ability of the competitive FET to quantify cortisol in a complex biological matrix.

These results confirm the feasibility of combining the competitive FET with the standard addition method for cortisol determination in real saliva. The relatively good agreement between the experimentally determined concentration and the expected concentration also indicates that the proposed approach can provide reliable measurements in the presence of the saliva matrix.

## 4. Conclusions

In this work, a competitive FET based on a Si_3_N_4_-gated sensor and anti-cortisol antibody-functionalized magnetic nanoparticles was developed for label-free cortisol detection by electrochemical impedance spectroscopy. To the best of our knowledge, this study represents the first demonstration of this MNP-assisted competitive FET approach for cortisol detection.

The sensor showed a concentration-dependent response over 0.02–0.6 ng/mL, with a limit of detection of 0.006 ng/mL, and demonstrated a stronger response toward cortisol than the investigated non-target biomarkers. The feasibility of the platform was further demonstrated using real saliva, with a measured cortisol concentration of 11.6 ng/mL compared with an expected value of 10.2 ng/mL, corresponding to a 113.7% recovery.

These preliminary results demonstrate the potential of the proposed platform for cortisol detection in biological samples. Further studies will focus on improving reproducibility and analytical validation using a larger number of saliva samples, with the long-term objective of developing a compact platform for point-of-care cortisol monitoring.

## Figures and Tables

**Table 1:** Fitting parameters obtained from the equivalent circuit model [R_s_ + C_dl_/R_ct_].

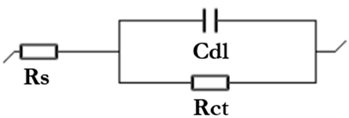	R_S_	C_dl_ · 10^9^ (F · S^a−1^)	R_ct_ · 10^6^ (Ohm)	X^2^ · 10^−3^
**Reference**	26.966	0.251	2.135	8.365
**0.6 ng mL^−1^**	21.029	0.254	2.599	2.472
**0.4 ng mL^−1^**	23.899	0.248	2.695	2.514
**0.2 ng mL^−1^**	28.859	0.264	2.967	5.513
**0.1 ng mL^−1^**	28.751	0.258	3.298	1.469
**0.04 ng mL^−1^**	31.816	0.254	3.722	4.008
**0.02 ng mL^−1^**	34.434	0.255	4.012	5.571
**MNPs-Ab**	37.816	0.257	4.935	4.651

**Table 2:** Comparison of different electrochemical immunosensors for cortisol detection.

Transducer	Sensing	Linear Range	LOD	Reference
Screen- printed carbon electrodes	Molecularly cortisol-imprinted acrylamide polymers	0.2–23.2 ng/mL	0.05 ng/mL	[35]
EG-FET	Pt/graphene/aptamer	0.4 ng/mL–0.4 μg/mL	0.007 ng/mL	[18]
rGO electrode	Ab/d-BSA/rGO	0.0036–36.2 ng/mL	0.036 ng/mL	[36]
Si_3_N_4_ FET	Ab/TESUD	0.01–0.6 ng/mL	0.005 ng/mL	[31]
Si_3_N_4_ FET	MNPs-Ab/Ag/TESUD	0.02–0.6 ng/mL	0.006 ng/mL	This work

## Data Availability

The data that support the findings of this study are available from the corresponding author upon reasonable request.

## References

[1] Kaur J., Gandhi J., Sharma S. (2026). Physiology, Cortisol. StatPearls.

[2] Buckingham J.C. (2006). Glucocorticoids: Exemplars of Multi-Tasking. Br. J. Pharmacol..

[3] Pivonello R., De Martino M.C., Iacuaniello D., Simeoli C., Muscogiuri G., Carlomagno F., De Leo M., Cozzolino A., Colao A., Arvat E., Falorni A. (2016). Metabolic Alterations and Cardiovascular Outcomes of Cortisol Excess. Frontiers of Hormone Research.

[4] Ortiz R., Kluwe B., Lazarus S., Teruel M.N., Joseph J.J. (2022). Cortisol and Cardiometabolic Disease: A Target for Advancing Health Equity. Trends Endocrinol. Metab..

[5] Inder W.J., Dimeski G., Russell A. (2012). Measurement of Salivary Cortisol in 2012—Laboratory Techniques and Clinical Indications. Clin. Endocrinol..

[6] Hellhammer D.H., Wüst S., Kudielka B.M. (2009). Salivary Cortisol as a Biomarker in Stress Research. Psychoneuroendocrinology.

[7] Suryawanshi R., Sharma S. (2026). Cortisol as a Stress Biomarker: Analytical Perspectives and Challenges. Clin. Chim. Acta.

[8] An J.E., Kim K.H., Park S.J., Seo S.E., Kim J., Ha S., Bae J., Kwon O.S. (2022). Wearable Cortisol Aptasensor for Simple and Rapid Real-Time Monitoring. ACS Sens..

[9] Wang B., Zhao C., Wang Z., Yang K.-A., Cheng X., Liu W., Yu W., Lin S., Zhao Y., Cheung K.M. (2022). Wearable Aptamer-Field-Effect Transistor Sensing System for Noninvasive Cortisol Monitoring. Sci. Adv..

[10] Vignesh V., Castro-Dominguez B., James T.D., Gamble-Turner J.M., Lightman S., Reis N.M. (2024). Advancements in Cortisol Detection: From Conventional Methods to Next-Generation Technologies for Enhanced Hormone Monitoring. ACS Sens..

[11] Karachaliou C.-E., Koukouvinos G., Goustouridis D., Raptis I., Kakabakos S., Petrou P., Livaniou E. (2023). Cortisol Immunosensors: A Literature Review. Biosensors.

[12] Turpeinen U., Hämäläinen E. (2013). Determination of Cortisol in Serum, Saliva and Urine. Best Pract. Res. Clin. Endocrinol. Metab..

[13] Kaushik A., Vasudev A., Arya S.K., Pasha S.K., Bhansali S. (2014). Recent Advances in Cortisol Sensing Technologies for Point-of-Care Application. Biosens. Bioelectron..

[14] Bergveld P. (1970). Development of an Ion-Sensitive Solid-State Device for Neurophysiological Measurements. IEEE Trans. Biomed. Eng..

[15] Bergveld P. (2003). Thirty Years of ISFETOLOGY. Sens. Actuators B Chem..

[16] Cao S., Sun P., Xiao G., Tang Q., Sun X., Zhao H., Zhao S., Lu H., Yue Z. (2023). ISFET-Based Sensors for (Bio)Chemical Applications: A Review. Electrochem. Sci. Adv..

[17] Sakata T. (2024). Signal Transduction Interfaces for Field-Effect Transistor-Based Biosensors. Commun. Chem..

[18] Sheibani S., Capua L., Kamaei S., Akbari S.S.A., Zhang J., Guerin H., Ionescu A.M. (2021). Extended Gate Field-Effect-Transistor for Sensing Cortisol Stress Hormone. Commun. Mater..

[19] Jang H.-J., Lee T., Song J., Russell L., Li H., Dailey J., Searson P.C., Katz H.E. (2018). Electronic Cortisol Detection Using an Antibody-Embedded Polymer Coupled to a Field-Effect Transistor. ACS Appl. Mater. Interfaces.

[20] Sesay A.M., Micheli L., Tervo P., Palleschi G., Virtanen V. (2013). Development of a Competitive Immunoassay for the Determination of Cortisol in Human Saliva. Anal. Biochem..

[21] Sun B., Gou Y., Ma Y., Zheng X., Bai R., Ahmed Abdelmoaty A.A., Hu F. (2017). Investigate Electrochemical Immunosensor of Cortisol Based on Gold Nanoparticles/Magnetic Functionalized Reduced Graphene Oxide. Biosens. Bioelectron..

[22] Apilux A., Rengpipat S., Suwanjang W., Chailapakul O. (2020). Paper-Based Immunosensor with Competitive Assay for Cortisol Detection. J. Pharm. Biomed. Anal..

[23] Hsing I., Xu Y., Zhao W. (2007). Micro- and Nano- Magnetic Particles for Applications in Biosensing. Electroanalysis.

[24] Xie L., Jiang R., Zhu F., Liu H., Ouyang G. (2014). Application of Functionalized Magnetic Nanoparticles in Sample Preparation. Anal. Bioanal. Chem..

[25] Moreno-Guzmán M., Eguílaz M., Campuzano S., González-Cortés A., Yáñez-Sedeño P., Pingarrón J.M. (2010). Disposable Immunosensor for Cortisol Using Functionalized Magnetic Particles. Analyst.

[26] Karaman C., Aydemir M., Karaman O. (2026). Wired for Stress: Advances in Electrochemical Biosensors for Monitoring of Cortisol and Catecholamines via Wearable and Point-of-Care Platforms. TrAC Trends Anal. Chem..

[27] Vozgirdaite D., Ben Halima H., Bellagambi F.G., Alcacer A., Palacio F., Jaffrezic-Renault N., Zine N., Bausells J., Elaissari A., Errachid A. (2021). Development of an ImmunoFET for Analysis of Tumour Necrosis Factor-α in Artificial Saliva: Application for Heart Failure Monitoring. Chemosensors.

[28] Ben Halima H., Bellagambi F.G., Alcacer A., Pfeiffer N., Heuberger A., Hangouët M., Zine N., Bausells J., Elaissari A., Errachid A. (2021). A Silicon Nitride ISFET Based Immunosensor for Tumor Necrosis Factor-Alpha Detection in Saliva. A Promising Tool for Heart Failure Monitoring. Anal. Chim. Acta.

[29] Ben Halima H., Bellagambi F.G., Hangouët M., Alcacer A., Pfeiffer N., Heuberger A., Zine N., Bausells J., Errachid A. (2023). A Novel IMFET Biosensor Strategy for Interleukin-10 Quantification for Early Screening Heart Failure Disease in Saliva. Electroanalysis.

[30] Ben Halima H., Bellagambi F.G., Hangouët M., Alcacer A., Pfeiffer N., Heuberger A., Zine N., Bausells J., Elaissari A., Errachid A. (2023). A Novel Electrochemical Strategy for NT-proBNP Detection Using IMFET for Monitoring Heart Failure by Saliva Analysis. Talanta.

[31] Ben Halima H., Bellagambi F.G., Brunon F., Alcacer A., Pfeiffer N., Heuberger A., Hangouët M., Zine N., Bausells J., Errachid A. (2023). Immuno Field-Effect Transistor (ImmunoFET) for Detection of Salivary Cortisol Using Potentiometric and Impedance Spectroscopy for Monitoring Heart Failure. Talanta.

[32] Ben Halima H., Zine N., Nemeir I.A., Pfeiffer N., Heuberger A., Bausells J., Elaissari A., Jaffrezic-Renault N., Errachid A. (2024). An ImmunoFET Coupled with an Immunomagnetic Preconcentration Technique for the Sensitive EIS Detection of HF Biomarkers. Micromachines.

[33] Bellagambi F.G., Degano I., Ghimenti S., Lomonaco T., Dini V., Romanelli M., Mastorci F., Gemignani A., Salvo P., Fuoco R. (2018). Determination of Salivary α-Amylase and Cortisol in Psoriatic Subjects Undergoing the Trier Social Stress Test. Microchem. J..

[34] Shrivastava A., Gupta V. (2011). Methods for the Determination of Limit of Detection and Limit of Quantitation of the Analytical Methods. Chron. Young Sci..

[35] Khan M.S., Dighe K., Wang Z., Srivastava I., Schwartz-Duval A.S., Misra S.K., Pan D. (2019). Electrochemical-Digital Immunosensor with Enhanced Sensitivity for Detecting Human Salivary Glucocorticoid Hormone. Analyst.

[36] Kim K.S., Lim S.R., Kim S.-E., Lee J.Y., Chung C.-H., Choe W.-S., Yoo P.J. (2017). Highly Sensitive and Selective Electrochemical Cortisol Sensor Using Bifunctional Protein Interlayer-Modified Graphene Electrodes. Sens. Actuators B Chem..

